# SWATH-MS-Based Proteomics Reveals the Regulatory Metabolism of Amaryllidaceae Alkaloids in Three *Lycoris* Species

**DOI:** 10.3390/ijms24054495

**Published:** 2023-02-24

**Authors:** Meng Tang, Chaohan Li, Cheng Zhang, Youming Cai, Yongchun Zhang, Liuyan Yang, Moxian Chen, Fuyuan Zhu, Qingzhu Li, Kehu Li

**Affiliations:** 1 Forestry and Pomology Research Institute, Protected Horticultural Research Institute, Shanghai Key Laboratory of Protected Horticultural Technology, Shanghai Academy of Agricultural Sciences, Shanghai 201403, China; 2 Co-Innovation Center for Sustainable Forestry in Southern China, College of Biology and the Environment, Nanjing Forestry University, Nanjing 210037, China; 3 Key Laboratory of Plant Resource Conservation and Germplasm Innovation in Mountainous Region (Ministry of Education), Collaborative Innovation Center for Mountain Ecology & Agro-Bioengineering (CICMEAB), Institute of Agro-Bioengineering, College of Life Sciences, Guizhou University, Guiyang 550025, China

**Keywords:** alkaloids, galanthamine, *Lycoris*, metabolism, SWATH-MS

## Abstract

Alkaloids are a class of nitrogen-containing alkaline organic compounds found in nature, with significant biological activity, and are also important active ingredients in Chinese herbal medicine. Amaryllidaceae plants are rich in alkaloids, among which galanthamine, lycorine, and lycoramine are representative. Since the difficulty and high cost of synthesizing alkaloids have been the major obstacles in industrial production, particularly the molecular mechanism underlying alkaloid biosynthesis is largely unknown. Here, we determined the alkaloid content in *Lycoris longituba*, *Lycoris incarnata*, and *Lycoris sprengeri*, and performed a SWATH-MS (sequential window acquisition of all theoretical mass spectra)-based quantitative approach to detect proteome changes in the three *Lycoris*. A total of 2193 proteins were quantified, of which 720 proteins showed a difference in abundance between *Ll* and *Ls*, and 463 proteins showed a difference in abundance between *Li* and *Ls*. KEGG enrichment analysis revealed that differentially expressed proteins are distributed in specific biological processes including amino acid metabolism, starch, and sucrose metabolism, implicating a supportive role for Amaryllidaceae alkaloids metabolism in *Lycoris*. Furthermore, several key genes collectively known as OMT and NMT were identified, which are probably responsible for galanthamine biosynthesis. Interestingly, RNA processing-related proteins were also abundantly detected in alkaloid-rich *Ll*, suggesting that posttranscriptional regulation such as alternative splicing may contribute to the biosynthesis of Amaryllidaceae alkaloids. Taken together, our SWATH-MS-based proteomic investigation may reveal the differences in alkaloid contents at the protein levels, providing a comprehensive proteome reference for the regulatory metabolism of Amaryllidaceae alkaloids.

## 1. Introduction

*Lycoris* spp. Amaryllidaceae is found in tropical and temperate regions, especially in China and Japan. It has long been widely used as folk medicine to treat various diseases [1,2]. *Lycoris* bulbs are used as traditional Chinese herbal medicine to treat sore throat, abscess, cancer, suppurative wound, poliomyelitis, mastitis, otitis media, ulcer, and neurodegenerative diseases [3]. According to the “Compendium of Chinese Materia Medica”, *Lycoris* also works in detoxifying, diminishing inflammation, pain relief, and diuresis [4]. Alkaloids are the main medicinal chemicals in the bulbs of *Lycoris* plants. Alkaloids are heterocyclic nitrogen compounds and have significant biological activities in human health applications. For example, the application in medicine is morphine, which has a powerful analgesic and sedative effect in clinical practice. Diterpenoid alkaloids isolated from Ranunculaceae were found to have antibacterial properties [5,6]. Carbohydrate alkaloids extracted from *Solanum nigrum* berries are medically used in the prevention of HIV infection and AIDS-related intestinal infections [7,8]. More than 600 alkaloids with various structures have been isolated from *Lycoris* plants including galanthamine, lycoramine, and lycorine thus far and have a variety of biological activities including anticancer, anti-inflammatory, anti-plasmodium, and antibacterial activities [3,9].

Galanthamine is a representative alkaloid of *Lycoris*, which is a selective, long-acting, reversible, and competitive acetylcholinesterase inhibitor. Symptomatic treatment for AD slows the progression of the disease and helps relieve memory loss [10]. Recently, transcriptomic and metabolomic analyses of *L. radiata* were performed to investigate the biosynthesis of galanthamine in different organs. It was found that *LrNNR*, *LrN4OMT,* and *LrCYP96T* were highly expressed in bulbs, which is consistent with the observation that more galanthamine is present in bulbs than in roots and leaves [11]. Functional characterization of the *lycoris* phenylalanine ammonia-lyase gene (*LrPAL*) revealed that *LrPAL* may be the limiting factor for the biosynthesis of galanthamine [12]. On the other hand, NpNBS condenses tyramine and 3,4-DHBA into norbelladine which is the first step in benzylisoquinoline alkaloid biosynthesis [13]. In addition, recombinant LlOMT catalyzes norbelladine to generate 4′-O-methylnorbelladine and overexpression of *LlOMT* in *Lycoris longituba* could increase the galanthamine content [14,15]. To date, previous research on the biosynthesis of galanthamine only focused on a few key genes, and the whole biosynthetic pathway of galanthamine in plants has not been comprehensively investigated particularly for the metabolism regulatory network from the perspective of proteome level.

This study was conducted by SWATH-MS technology to explore the proteome of *Lycoris*. SWATH-MS is a specific data-independent-acquisition (DIA)-based method and is emerging as a technology that combines deep proteome coverage capabilities with quantitative consistency and accuracy [16]. SWATH overcomes the problem of stochasticity in data-dependent acquisition (DDA), making the detection results more reproducible and consistent. The wider coverage and higher detection sensitivity will further provide more ways to verify the function of the target protein in plants, which makes SWATH-MS a huge prospect in plant proteomics research [17]. In this study, the relative quantitative comparison of proteins among three groups of *Lycoris* samples with different alkaloid contents was carried out by quantitative proteomics technology based on SWATH-MS, and a total of 2193 proteins were detected. The differentially expressed proteins among the three groups were enriched in the amino acid metabolism pathway, galanthamine synthesis pathway, and sucrose metabolism pathway. Analysis of bioinformatics data will offer considerable information on the proteome among the three *Lycoris* with different alkaloid contents, and also provide deeper insights into the molecular mechanisms of Amaryllidaceae alkaloid’s regulatory metabolism. In summary, our SWATH-MS-based proteomics study can provide new insights into the mechanism of alkaloid-regulated metabolism in Amaryllidaceae at the protein level, and provide new ideas for the yield enhancement of alkaloid biosynthesis in the future.

## 2. Results

### 2.1. SWATH-MS-Based Proteomic Analysis among Three Species of Lycoris with Different Alkaloid Content

**Figure 1:**
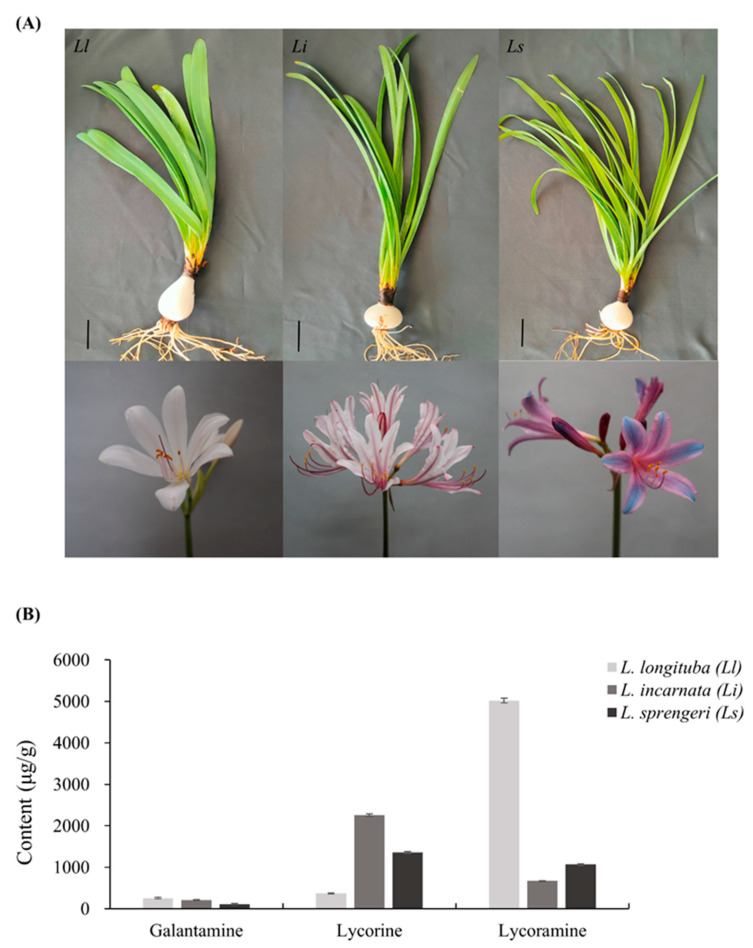
Phenotype of samples and alkaloid contents of three kinds of *Lycoris* species. (**A**) The three kinds of plant materials: *Lycoris longituba*, *Lycoris incarnata*, and *Lycoris sprengeri*. They were collected from the Institute of Botany, Chinese Academy of Sciences, Jiangsu Province, China (32.05° N, 118.83° E) in late March when the leaves were growing vigorously (Bars = 3 cm). (**B**) The total content of three alkaloids in *Lycoris longituba* was the highest, whereas that in *Lycoris sprengeri* was the lowest.

To further investigate the biosynthesis of Amaryllidaceae alkaloids in *Lycoris* species, we performed a SWATH-MS-based proteomic analysis of *Lycoris longituba* (*Ll*), *Lycoris incarnata* (*Li*), and *Lycoris sprengeri* (*Ls*) with different alkaloid contents. The three *Lycoris* materials were collected from the Institute of Botany, Chinese Academy of Sciences, Jiangsu Province, China. Among them, the total content of alkaloids in *Ll* was the highest, whereas in *Ls* was the lowest (Figure 1B).

**Figure 2:**
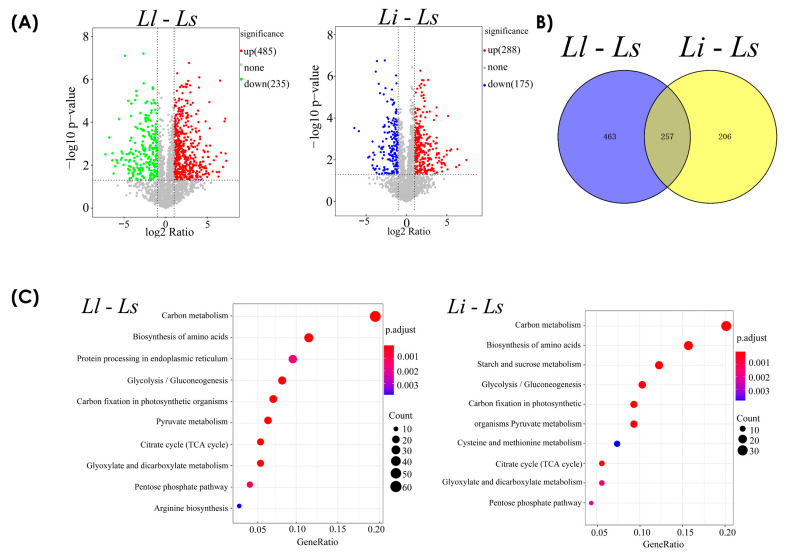

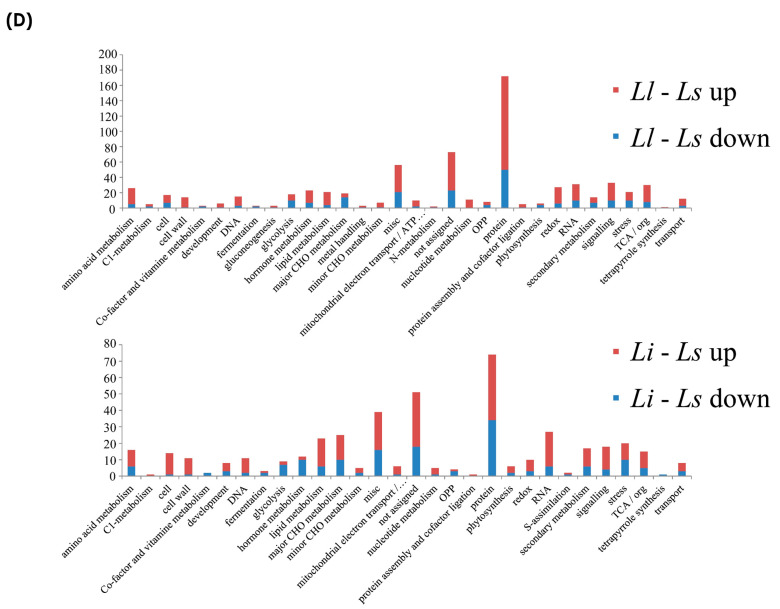
The overview and functional classification by KEGG and MAPMAN BIN.of DEPs. (**A**) The volcano picture shows the distribution of differentially expressed proteins with different fold changes. (**B**) Venn diagram shows the number of differential proteins compared between the two groups. (**C**) KEGG pathway classification was performed on the differential proteins of the 2 groups and enriched in the top 10 pathways. Amino acid biosynthesis, starch, and sucrose metabolic pathways are significantly activated. (**D**) MAPMAN BIN categorization of DEPs; blue and red represent down-regulated and up-regulated proteins, respectively.

We selected three groups of samples as biological replicates for proteomic analysis. The correlation coefficient of the samples within the group and the principal component clustering results strongly reflected the high reliability of the appropriate sampling and suitability for subsequent analysis (Figure S1A). A total of 2193 proteins were quantified, and the group with the lowest alkaloid content *Ls* served as the control group. Proteins with a fold change above 2 or below 0.5 (*p* < 0.05) were considered differentially expressed proteins (DEPs) in this study. As shown in the volcano diagram, 485 upregulated proteins and 235 downregulated proteins were detected in *Ll* compared with *Ls*, whereas 288 upregulated proteins and 175 downregulated proteins were detected in *Li* (Figure 2A). The cluster heatmap showed significant changes in protein abundance. When we compared *Ll* with *Ls*, red and green colors, respectively, indicated upregulation and downregulation; when we compared *Li* with *Ls*, red and blue colors, respectively, indicated upregulation and downregulation (Figure S1C,D). This suggests that some crucial regulators or pathways have been induced, potentially affecting the biosynthesis of Amaryllidaceae alkaloids in *Lycoris*.

### 2.2. Functional Classification of DEPs

KEGG pathway classification was performed on the two groups’ differential proteins and enriched in the top 10 pathways in order to quickly view the pathways that affect the biosynthesis of Amaryllidaceae alkaloids in *Lycoris*. For example, amino acid biosynthesis and starch and sucrose metabolic pathways were significantly activated and may be involved in contributing to the biosynthesis of Amaryllidaceae alkaloids in *Lycoris* (Figure 2C). Subsequently, we used the MapMAN BIN system to functionally classify the differentially expressed proteins into two groups. For example, compared with *Ls*, all differential proteins were assigned to 32 functional categories in *Ll*, including “protein metabolism”, “RNA processing and transport”, “signal transduction”, and other categories which constitute the main part of differential proteins, indicating these proteins have a great impact on the biosynthesis of Amaryllidaceae alkaloids in *Lycoris*. Among these, 21 of the 26 proteins involved in amino acid metabolism were downregulated. In the following sections, these upregulated or downregulated proteins will be mapped to different physiological and biochemical pathways, and the correlation between these biochemical pathways and the biosynthesis of alkaloids will be discussed (Figure 2D).

### 2.3. Effects of Amino Acid Metabolism on the Biosynthesis of Amaryllidaceae Alkaloids in Lycoris

Amino acids are closely related to the synthesis of plant alkaloids, and the nitrogen in complex alkaloids comes from amines derived from amino acid metabolism [18]. The amines contributing to alkaloid biosynthesis are derived from various amino acids, and are divided into two categories including polyamines and aromatic amines. Tyrosine is the precursor of multiple alkaloid families, including benzylisoquinolines (BIAs), Amaryllidaceae alkaloids, and betalains. *TyrDC* from Amaryllidaceae alkaloid biosynthesis has recently been discovered, it enables the incorporation of tyramine into the structures [15].

**Figure 3:**
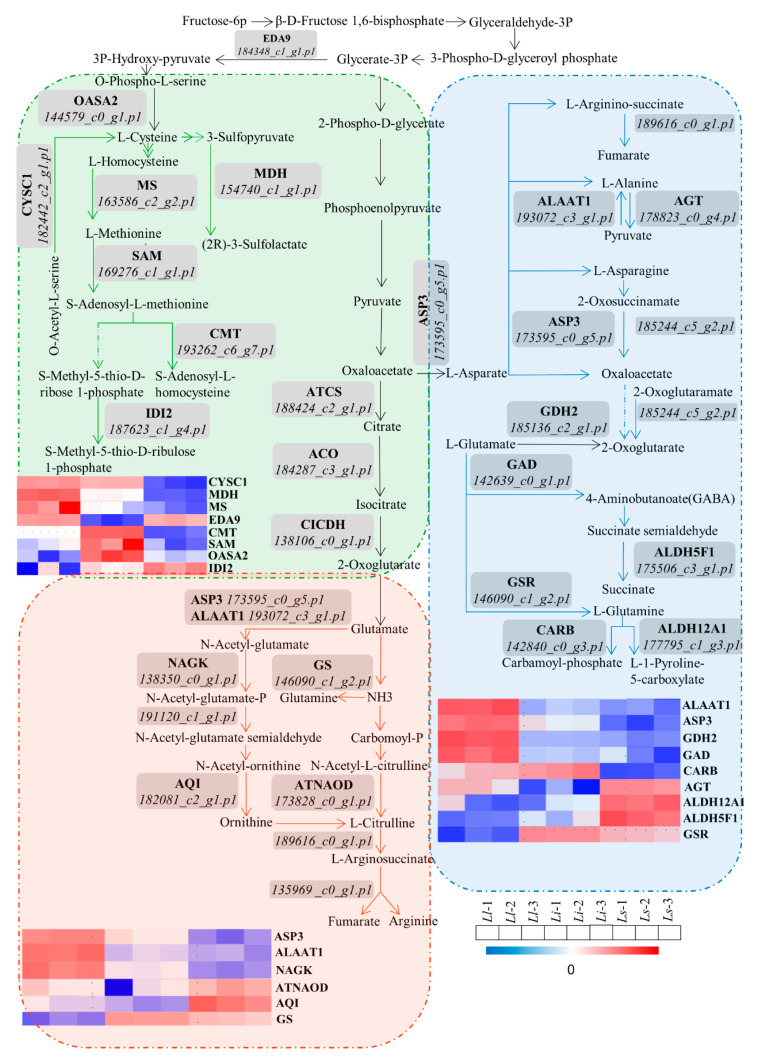
Amino acid metabolism is closely related to the biosynthesis of Amaryllidaceae alkaloids in *Lycoris*. We detected a total of 38 differential proteins in the amino acid metabolism pathway. The highly expressed proteins were mainly concentrated in Ll, which was consistent with the actual detected trend of alkaloid content of the three varieties.

Through previous analyses of KEGG and MAPMAN BIN, we integrated the identified proteins embedded into different amino acid biosynthesis pathways by KEGG and MAPMAN BIN analysis (Table S1). Proteins with significant changes in abundance were enriched in the biosynthesis of alanine, aspartate, glutamate, cysteine, methionine, and arginine (Figure 3), of which alanine and arginine are the raw materials for the biosynthesis of plant alkaloids. Conversely, various alkaloids can negatively regulate the biosynthesis of glutamate in cells [19].

We detected a total of 38 differential proteins in the amino acid metabolism pathway, mainly concentrated in the biosynthesis and metabolism pathways of amino acids such as alanine, arginine, and cysteine. Among them, 18 genes were upregulated in *Ll*, 12 genes were upregulated in *Li*, and 8 genes were upregulated in *Ls*. In general, the highly expressed proteins were mainly concentrated in *Ll*, which was consistent with the highest alkaloid content of *Ll*, implicating that amino acid metabolism is closely related to the biosynthesis of Amaryllidaceae alkaloids in *Lycoris*.

### 2.4. Important Genes Involved in the Galanthamine Biosynthesis Pathway for Alkaloids Production in Lycoris

**Figure 4:**
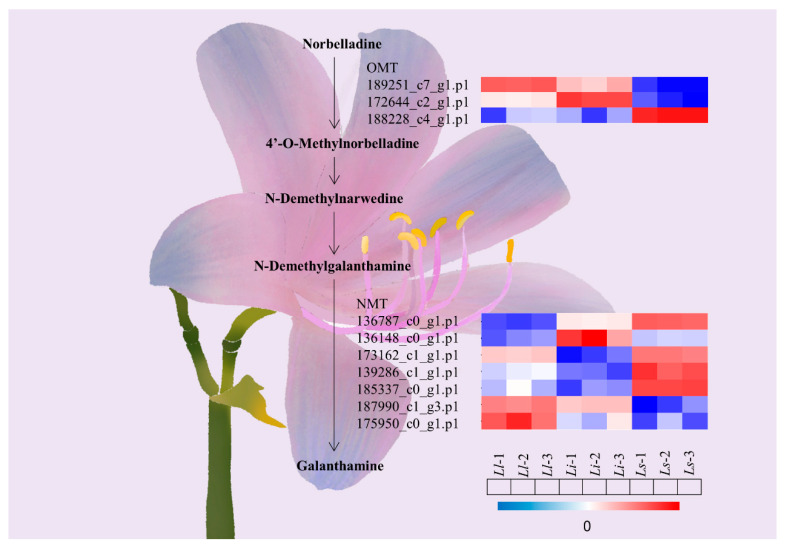
Candidate genes responsible for the biosynthesis of galanthamine in *Lycoris*. 189251_c7_g1.p1 and 175950_c0_g1.p1 and other genes upregulated in *Ll*, which provide the raw material for the biosynthesis of galanthamine, consistent with the highest galanthamine content in Ll.

Galanthamine is a unique isoquinoline alkaloid and competitive acetylcholinesterase inhibitor that has broad prospects for future medical applications. The difficulty and high cost of synthesizing galanthamine have been the major obstacles to industrial production. The biosynthetic pathway of Gal has recently been elucidated including phenylalanine ammonia lyase (PAL), cinnamic acid-4-hydroxylase (C4H), coumaric acid 3-hydroxylase, tyrosine decarboxylase (TYDC), desmethylbelladine synthase (NBS), desmethylbelladine 40-O-methyltransferase (OMT), desmethoxymalidine synthase (CYP96T1), and N-methyltransferase (NMT) [15,20]. *OMT* is a methyltransferase that is involved in the biosynthesis of many alkaloids. For example, *OMT* has been shown to be responsible for multiple substrate and region-specific methylation in the roots of *G. flavum* and the biosynthesis and metabolism of plant alkaloids [21,22]. *NMT* is also a methyltransferase that has been shown to catalyze phenylisoquinoline alkaloids, one biosynthetic precursor of morphine [23]. In this study, we detected ten differentially expressed proteins among the three *Lycoris* species in the biosynthetic pathway of galanthamine. Among them, three proteins collectively known as OMT probably catalyzed norbelladine into 4′-O-methylnorbelladine and subsequently oxidized to N-demethylnawedine, further reduced to N-demethylgalanthamine. Seven other identified proteins known as NMT are probably responsible for the methylation of N-demethylgalanthamine to galanthamine (Figure 4).

### 2.5. Other Pathways Related to the Amaryllidaceae alkaloids in Lycoris

**Figure 5:**
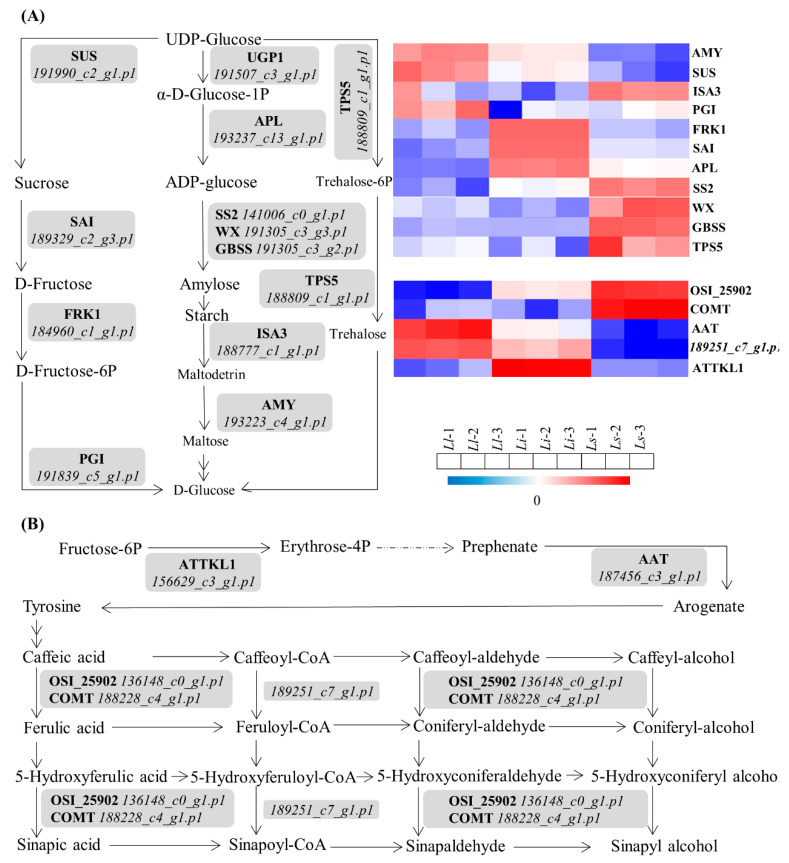
Potenial pathways contribute to the biosynthesis of Amaryllidaceae alkaloids in *Lycoris*. (**A**) Some proteins were detected to be enriched in starch and sucrose metabolism pathways suggesting a reciprocal regulation between alkaloids and sugar metabolism. (**B**) In secondary metabolic pathways, the up-regulated 187456_c3_g1.p1 and 189251_c7_g1.p1 in Ll participate in the biosynthesis of sinapyl alcohol, resulting in an increase in the alkaloid raw material sinapyl alcohol. Eventually, this leads to a rise in alkaloid content.

According to our MapMAN BIN system results, some proteins were detected to be enriched in starch and sucrose metabolism pathways suggesting a reciprocal regulation between alkaloids and sugar metabolism on the seasonal variation of alkaloids and total polyphenol contents, showing a contrasting tendency in sugar content and tissue development [24] (Figure 5A). Likewise, some proteins showed differences in abundance in secondary metabolic pathways, which are likely to contribute to the biosynthesis of alkaloids. For example, the upregulated *187456_c3_g1.p1* and *189251_c7_g1.p1* in *Ll* participate in the biosynthesis of sinapyl alcohol, resulting in an increase in the alkaloid raw material sinapyl alcohol, which would eventually lead to a rise in alkaloid content [25] (Figure 5B).

## 3. Discussion

### 3.1. Advanced High-Throughput Omics Technology Facilities the Elucidation on the Regulatory Metabolism of Lycoris Species

Multiple-omics technologies have been extensively applied to various aspects of *Lycoris* research. Transcriptome analysis facilities the identification of key regulatory genes involved in anthocyanin metabolism during flower development in *Lycoris radiata* [26]. Proteomic analysis helps us better understand the molecular mechanism of *L. radiata* development and provides valuable information about the proteins involved in the development and stress response of other *Lycoris* genera [27]. Multi-omics such as transcriptomic and metabolomic analyses reveal that exogenous methyl jasmonate regulates galanthamine biosynthesis in *Lycoris longituba* seedlings; furthermore, anthocyanin biosynthesis, steroid biosynthesis, and R2R3 MYB TFs may play vital regulatory roles in petal color development in *L. sprengeri* [15,28]. Therefore, we used innovative SWATH-MS quantitative proteomics to investigate the molecular regulatory network of Amaryllidaceae alkaloids metabolism in *Lycoris*, which provides a new strategy for the future exploration of the biosynthesis and function of secondary metabolites. Furthermore, single-cell-based omics reveals that alkaloids are localized in *Catharanthus roseus* stem and leaf tissues [29], which would be an effective strategy to determine intercellular localization of alkaloids from different tissues in *Lycoris*, thus understanding the mechanism of alkaloids biosynthesis at the cellular level. A flow cytometry study of the nuclear DNA contents of *Lycoris* species (Amaryllidaceae) with different chromosome numbers revealed that the *Lycoris* genome contains approximately 30 G [30]. Since the *Lycoris* genome is so large and undiscovered, Pacbio’s new revolutionary long-read length sequencing system, revio, will significantly reduce sequencing costs, and is expected to be applied to the *Lycoris* genome, thus providing a high-quality map of the *Lycoris* genome and providing a scientific basis for the metabolic regulatory mechanisms in *Lycoris*.

### 3.2. Candidate Genes Responsible for the Biosynthesis of Amaryllidaceae alkaloids in Lycoris

Seeking some target proteins among the differentially expressed proteins is necessary to illuminate the biosynthesis process of Amaryllidaceae alkaloids in *Lycoris*. Here, we collected five candidate proteins with dramatically increasing abundances (fold changes > 100), as shown in Table 1. Representatively, non-specific lipid-transfer protein 3 (*LTP3: TRIBITY_DN163977_c0_g2.p1*) exhibited 118.2-fold upregulation in *Ll* and 173.7-fold upregulation in *Li*. Plant lipid transfer proteins (LTPs) exhibit the ability to transfer lipids between membranes in vitro and have been shown to promote the production of abscisic acid (ABA) production, thereby stimulating the accumulation of alkaloids in cells [31]. Interestingly, crosstalk was detected between the JA and abscisic acid (ABA) signaling pathways in the regulation of tobacco (Nicotiana tabacum) alkaloid biosynthesis [32]. Therefore, further research is needed to explore secondary metabolites which can regulate the biosynthesis of Amaryllidaceae alkaloids through the ABA signaling pathway. In short, further genetic and molecular strategies with bioinformatics analysis will confirm the roles of these candidate proteins related to the biosynthesis of Amaryllidaceae alkaloids in *Lycoris*.

### 3.3. RNA Processing Related Proteins May Contribute to the Biosynthesis of Amaryllidaceae alkaloids in Lycoris

RNA processing includes mRNA capping, splicing, cleavage, and polyadenylation, which participates in various key cellular processes and probably plays an important role in the biosynthesis of alkaloids. It was recently discovered that two potent anticancer alkaloids SANG and CHEL could directly bind to single-stranded RNAs, which reveals the fundamental structural and calorimetric aspects of the interaction of the natural benzophenanthridine alkaloids with single-stranded RNAs, facilitating the development of next-generation alkaloid therapeutics targeting single-stranded RNA [33]. Coincidentally, the polyadenylate [poly(A)] tail of mRNA was also found to have recently been identified as a potential drug target due to its important role in translational initiation, maturation, and stabilization of mRNA, and production of alternative proteins in eukaryotic cells. Some small molecular alkaloids with isoquinoline groups can bind to poly A with high affinity to form self-structures. It provides a reference for the development of novel bio-base molecules targeting poly(A) structures [34].

Eukaryotic pre-mRNAs are spliced to form mature mRNA. Alternative splicing greatly expands the transcriptomic and proteomic diversities related to physiological and developmental processes in higher eukaryotes. Alternative splicing is a posttranscriptional regulatory mechanism that generates multiple protein isoforms from a single gene through the use of alternative splice sites during splicing. However, the biosynthesis of alkaloids is a special metabolic pathway in plants, in which many genes have alternative splicing at different developmental stages and under stress conditions [35]. The finding of PR3b splicing regulation by JA/ET and NIC loci in Burley 21 is valuable to the genetic studies of low-alkaloid mutants and could provide clues to unravel the mechanisms by which JA/ET-signaling pathways regulate PR protein gene splicing [36]. Homoharringtonine (HHT) is a natural alkaloid with potent antitumor activity, which regulates the alternative splicing of Bel-x and Caspase 9 through a PP1-dependent mechanism, revealing a novel mechanism underlying the antitumor activities of HHT [37]. However, its role in the biosynthesis of galanthamine remains unclear. Interestingly, we found nine differentially expressed proteins related to RNA processing in the tested data in Table 2. This indicates that RNA processing is an important step in the biosynthesis of Amaryllidaceae alkaloids in *Lycoris*, and further experimental investigations are needed for functional validation.

## 4. Materials and Methods

### 4.1. Plant Materials

Plant materials of the three *Lycoris* varieties *Lycoris longituba*, *Lycoris incarnata*, and *Lycoris sprengeri* were collected from the Institute of Botany, Chinese Academy of Sciences, Jiangsu Province, China (32.05° N, 118.83° E) in late March when the leaves were growing vigorously (Figure 1A). Voucher specimens of *Lycoris longituba* (0653969), *Lycoris incarnata* (0653959), and *Lycoris sprengeri* (0653967) were deposited at the herbarium in the Institute of Botany, Chinese Academy of Sciences. The bulb samples with similar diameters and specifications to the three *Lycoris* varieties were selected, chopped, mixed well, and frozen at −70 °C.

### 4.2. Protein Extraction for Proteomic Analysis

Tissue samples were first ground into a powder with liquid nitrogen and added to an appropriate volume of lysis buffer (2.5% SDS/100 mM Tris-HCl, pH = 8.5), sonicated in an ice-water bath for 15 min and centrifuged at 16,000× *g* for 20 min until clarified. The protein in the supernatant was precipitated by the acetone method. After washing with acetone and drying, 8 M urea/100 mM Tris-HCl solution (pH 8.0) was added to the protein precipitate to fully dissolve the protein. The samples were centrifuged at 12,000× *g* for 15 min, the supernatant was collected, dithiothreitol (DTT) was added to a final concentration of 10 mM, and the samples were incubated at 37 °C for 1 h to perform a reduction reaction to open disulfide bonds. Then, iodoacetamide (IAA) was added to a final concentration of 40 mM, and an alkylation reaction was performed at room temperature in the dark to block sulfhydryl groups. An appropriate volume of 100 mM Tris-HCl solution (pH 8.0) was added, the Bradford method was used to quantify the protein concentration, the urea concentration was diluted to below 2 M, and trypsin was added to each of the samples according to the ratio of protein amount trypsin amount =50:1, and incubated at 37 °C overnight with shaking. The next day, TFA was added to terminate the digestion and the pH value of the solution was adjusted to approximately 6.0. The solution was centrifuged at 12,000× *g* for 15 min and a homemade C18 cartridge was used for desalting. The desalted peptide solution was dried by a centrifugal concentrator and then stored frozen at −20 °C for on-board detection.

### 4.3. SWATH-MS Analysis

The SWATH methods were used for subsequent MS-analysis using Triple TOF 5600 (Sciex) LC/MS system. The prepared peptide samples were first bound to the trap column and then separated by the analytical column (45 min gradient, 60 min total duration). Two mobile phases used to establish the analytical gradient were buffer A-0.1% (*v*/*v*) formic acid, 5% DMSO in H_2_O, buffer B-0.1% (*v*/*v*) formic acid, and 5% DMSO in acetonitrile. During SWATH scanning, each scan cycle consisted of one MS1 scan (ion accumulation time 250 ms, scan range 350–1500 *m*/*z*) and 100 variable window MS2 scans (ion accumulation time 33 ms, scan range 100–1800 *m*/*z*).

### 4.4. Alkaloid Extraction and Quantification

Galanthamine, lycoramine and lycorine were purchased from Shanghai TCI Development Co. Their characteristics have been described previously [38]. A total of 0.2 g of bulb samples of *Lycoris longituba*, *Lycoris incarnata*, and *Lycoris sprengeri* were taken to be tested, freeze-dried, and ground into powder. Extraction was performed by sonication with 2 mL ethanol (70% high-performance liquid chromatography (HPLC)–grade) for 30 min. After centrifugation at 12,000 rpm for 10 min, the supernatant was taken and dried under vacuum. The samples were redissolved in 1 mL 0.1% formic acid-acetonitrile (*v*/*v* = 95/5) for liquid phase analysis [15]. Waters ACQUITY UPLC BEH C18 column (150 mm × 2.1 mm, 1.7 μm) was used as the liquid chromatography column and the separation was conducted using 0.1% formic acid (*v*/*v*) (A) and acetonitrile (B) with a 6-min linear gradient of 5–60% B at a flow rate of 0.2 mL/min. Quantification of galanthamine, lycoramine, and lycorine used 288 → 231, 290 → 189, 288 → 146 (*m*/*z*) transition reactions respectively. Experiments were conducted with three independent biological replicates. Least-significant difference test (LSD, *p* < 0.05) was used to compare the means. Different letters represent significant differences between groups (*n* = 3, *p* < 0.05).

### 4.5. Data Analysis

The mass spectrum files obtained by SWATH scanning are processed by DIA-Umpire to obtain secondary mass spectrum files that are used for database search. TPP software, Comet, and X!tandem search engines were used to search the database, and the search results were used as the spectral library for subsequent target extraction. The algorithm used for SWATH targeted extraction quantification is OpenSWATH. The test results were screened with 1% FDR.

The protein quantitative intensity information obtained by SWATH analysis was used for difference comparison and T-test analysis after log2 transformation, data filling (imputation algorithm in Perseus software), and data normalization. Differential proteins were screened by fold difference (Ratio) and BH-corrected *p*-value (*P*.adjust). Proteins with a fold change above 2 or below 0.5 (*p* < 0.05) were considered differentially expressed proteins (DEPs) in this study. In the bioinformatics analysis, the DEPs identified were used for the Kyoto Encyclopedia of Genes and Genomes (KEGG) enrichment analysis by the clusterProfiler R package, and pathways of KEGG enrichment analysis results were drawn with reference to the KEGG mapper [39]. After the DEPs were compared with the homologous proteins in Arabidopsis thaliana, the MapMAN BIN system was used for functional classification.

## 5. Conclusions

Amaryllidaceae plants are rich in alkaloids such as galanthamine (Gal), lycoramine (Lycm), and lycorine (Lyc) with a variety of biological activities, including anticancer, anti-inflammatory, anti-plasmodium, and antibacterial activities. In this study, three alkaloids profiling of *Lycoris longituba* (*Ll*), *Lycoris incarnata* (*Li*), and *Lycoris sprengeri* (*Ls*) were determined, and a comprehensive proteomic analysis was carried out with the aim of investigating the regulatory metabolism of Amaryllidaceae alkaloids in *Lycoris* species. The significant proteome changes in amino acid metabolism, starch, and sucrose metabolism, and galanthamine biosynthesis strengthen our understanding of the regulatory metabolism of Amaryllidaceae alkaloids in *Lycoris*. Additionally, we have also identified important candidate genes involved in the galanthamine biosynthesis pathway of alkaloid production in *Lycoris*. Taken together, we have offered new thoughts on the use of SWATH-MS to explore the regulatory metabolism of Amaryllidaceae alkaloids, providing a new strategy for the future exploitation of alkaloids.

## Figures and Tables

**Table 1:** Some candidate genes responsible for the biosynthesis of Amaryllidaceae alkaloids in *Lycoris*.

Protein ID	Homolog-Arabidopsis	Description	Fold Change (*Ll*/*Ls*) (*p* < 0.05)	Fold Change (*Li*/*Ls*) (*p* < 0.05)
TRINITY_DN183542_c1_g2.p1	AT3G05500	Rubber elongation factor protein (REF)	153.6	21.0
TRINITY_DN147476_c3_g1.p1	AT1G08080	Alpha carbonic anhydrase 7	145.7	0.5
TRINITY_DN182379_c0_g1.p1	AT1G61490	S-locus lectin protein kinase family protein	142.6	312.8
TRINITY_DN163977_c0_g2.p1	AT5G59320	Non-specific lipid-transfer protein 3	118.2	173.7
TRINITY_DN194079_c6_g2.p1	AT5G59320	Non-specific lipid-transfer protein 3	1.2	330.9

**Table 2:** List of RNA processing-related proteins in DEPs.

Protein Name	Homolog-Arabidopsis	Fold Change	*p* Value	Functional Classification	Description
TRINITY_DN183625_c1_g1.p1	AT1G09760	2.054	0.005	RNA.processing	U2 small nuclear ribonucleoprotein A (U2A′)
TRINITY_DN183625_c1_g2.p1	AT1G09760	2.106	0.003	RNA.processing	U2 small nuclear ribonucleoprotein A (U2A′)
TRINITY_DN171323_c3_g1.p1	AT4G24770	12.734	0.008	RNA.processing.editing	31-kDa RNA binding protein (RBP31)
TRINITY_DN191152_c4_g3.p1	AT4G34110	7.243	0.035	RNA.processing	poly(A) binding protein 2 (PAB2)
TRINITY_DN171323_c3_g1.p1	AT4G24770	7.100	0.010	RNA.processing.editing	31-kDa RNA binding protein (RBP31)
TRINITY_DN183625_c1_g1.p1	AT1G09760	2.075	0.028	RNA.processing	U2 small nuclear ribonucleoprotein A (U2A′)
TRINITY_DN178056_c1_g3.p1	AT4G25630	2.001	0.002	RNA.processing	fibrillarin 2 (FIB2)
TRINITY_DN178691_c2_g1.p1	AT2G33430	0.018	0.008	RNA.processing.editing	Multiple organellar RNA editing factor 2, chloroplastic (MORF2)
TRINITY_DN188290_c2_g1.p1	AT2G47640	0.423	0.021	RNA.processing	Small nuclear ribonucleoprotein family protein (AT2G47640)
TRINITY_DN188183_c1_g1.p1	AT1G43190	0.086	0.042	RNA.processing	polypyrimidine tract-binding protein 3 (PTB3)
TRINITY_DN183238_c1_g2.p1	AT1G24450	0.269	0.001	RNA.processing.ribonucleases	Ribonuclease III family protein (NFD2)

## Data Availability

The data presented in this study are available upon request. The MS data have been deposited to the ProteomeXchange Consortium via the iProX repository with the data set identifier PXD040341.

## Supplementary Materials

The following supporting information can be downloaded at: https://www.mdpi.com/article/10.3390/ijms24054495/s1.
