# Supplementary material for: SWATH-MS-Based Proteomics Reveals the Regulatory Metabolism of Amaryllidaceae Alkaloids in Three Lycoris Species
Source: Int J Mol Sci. 2023 Feb 24;24(5):4495. doi: 10.3390/ijms24054495 (PMC10002500; doi:10.3390/ijms24054495)
Supplement: Supplementary file 1 [file ijms-24-04495-s001.zip › supplementary figures.pdf]

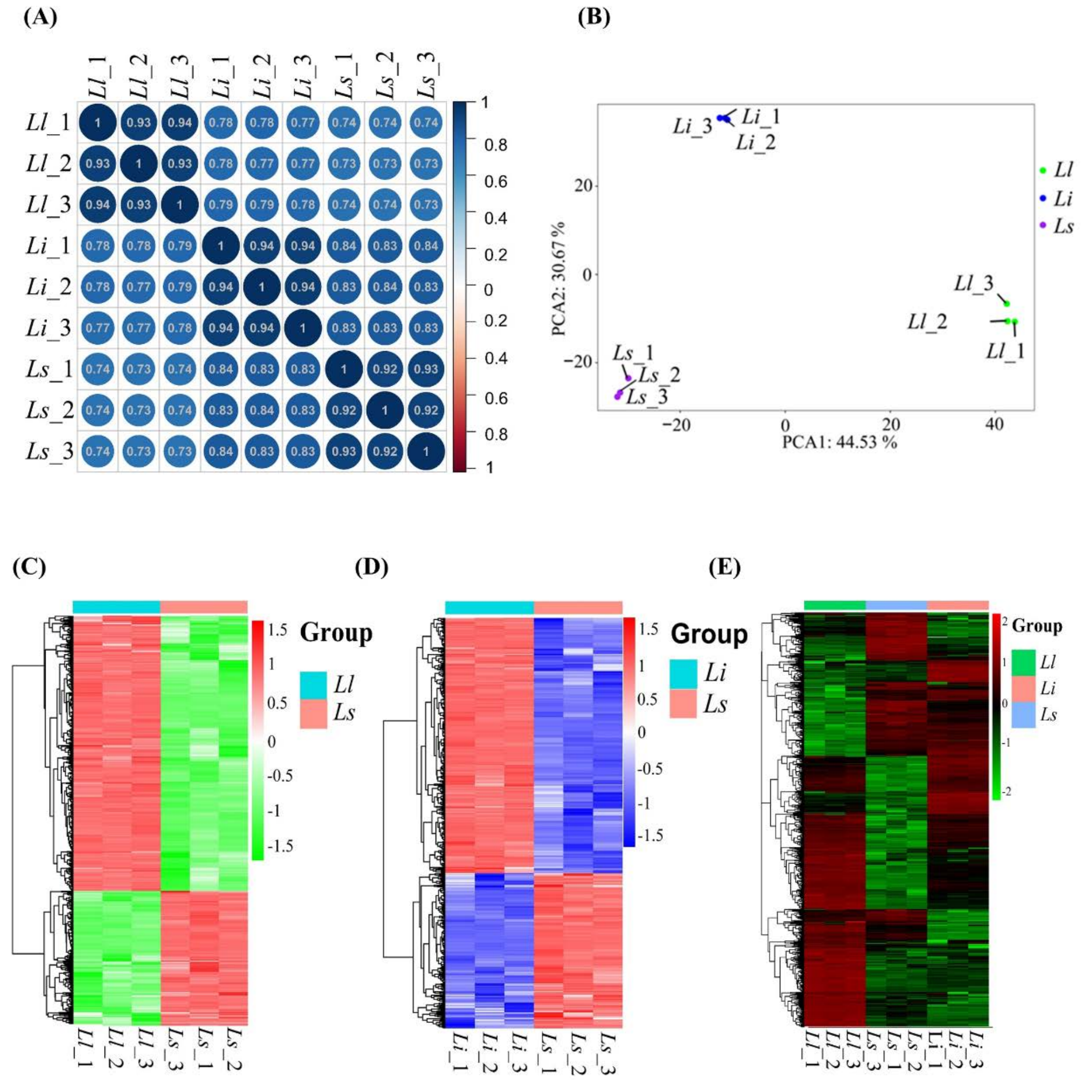

Figure S1. Summary of differential protein data. (A). The correlation diagram shows that the correlation of samples within the group is greater than 0.92, and the correlation of samples between the groups is also between 0.73 and 0.84. **(B).** Principal component analysis diagram. **(C-E).** Clustering heat maps showed significant changes in protein abundance.
